# The role of Nrf2 signaling in counteracting neurodegenerative diseases

**DOI:** 10.1111/febs.14379

**Published:** 2018-01-29

**Authors:** Albena T. Dinkova‐Kostova, Rumen V. Kostov, Aleksey G. Kazantsev

**Affiliations:** ^1^ Division of Cancer Research School of Medicine University of Dundee UK; ^2^ Departments of Medicine and Pharmacology and Molecular Sciences Johns Hopkins University School of Medicine Baltimore MD USA; ^3^ Department of Neurology Massachusetts General Hospital and Harvard Medical School Boston MA USA; ^4^ Present address: Effective Therapeutics Cambridge MA USA

**Keywords:** neurodegeneration, neuroinflammation, neuroprotection, Nrf2 activator, oxidative stress

## Abstract

The transcription factor Nrf2 (nuclear factor‐erythroid 2 p45‐related factor 2) functions at the interface of cellular redox and intermediary metabolism. Nrf2 target genes encode antioxidant enzymes, and proteins involved in xenobiotic detoxification, repair and removal of damaged proteins and organelles, inflammation, and mitochondrial bioenergetics. The function of Nrf2 is altered in many neurodegenerative disorders, such as Huntington's disease, Alzheimer's disease, amyotrophic lateral sclerosis, and Friedreich's ataxia. Nrf2 activation mitigates multiple pathogenic processes involved in these neurodegenerative disorders through upregulation of antioxidant defenses, inhibition of inflammation, improvement of mitochondrial function, and maintenance of protein homeostasis. Small molecule pharmacological activators of Nrf2 have shown protective effects in numerous animal models of neurodegenerative diseases, and in cultures of human cells expressing mutant proteins. Targeting Nrf2 signaling may provide a therapeutic option to delay onset, slow progression, and ameliorate symptoms of neurodegenerative disorders.

**Abbreviations**
AD: Alzheimer's disease
ALS: amyotrophic lateral sclerosis
AMPK: AMP‐activated protein kinase
APP: amyloid precursor protein
ARE/EpRE: antioxidant/electrophile response element
Aβ: amyloid‐β
CAT: catalase
ERK: extracellular signal‐regulated kinases
FRDA: Friedreich's ataxia
FTLD: frontotemporal lobar degeneration
GPX: glutathione peroxidase
GSH: reduced glutathione
GSK3: glycogen synthase kinase 3
GST: glutathione *S*‐transferase
HD: Huntington's disease
HFD: high‐fat diet
HO‐1: heme oxygenase 1
HTT: huntingtin
IL: interleukin
Keap1: Kelch‐like ECH‐associated protein 1
MPP+: positively charged 1‐methyl‐4‐phenylpyridinium
MPTP: 1‐methyl‐4‐phenyl‐1, 2, 3, 6‐tetrahydropyridine
NQO1: NAD(P)H: quinone oxidoreductase 1
Nrf2: nuclear factor‐erythroid 2 p45‐related factor 2
PD: Parkinson's disease
PGC‐1α: proliferator‐activated receptor gamma coactivator 1α
ROS: reactive oxygen species
SCF: Skp, Cullin, F‐box containing complex
SOD1: superoxide dismutase 1
tBHQ: *tert*‐butyl‐hydroquinone
TNF‐α: tumor necrosis factor alpha

## Introduction

**Figure 1:**
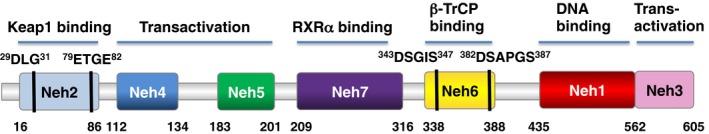
Domain structure of Nrf2. The low affinity binding ‘DLG’ motif and the high affinity binding ‘ETGE’ motif in the N‐terminal Neh2 domain, through which Nrf2 binds to Keap1, are indicated. Also shown is the phosphodegron in the Neh6 domain through which, following phosphorylation by glycogen synthase kinase 3 (GSK3), Nrf2 binds to β‐transducin repeat‐containing protein (β‐TrCP).

The transcription factor Nrf2 (nuclear factor erythroid 2 p45‐related factor 2) regulates the expression of genes involved in cellular protection against damage by oxidants, electrophiles, and inflammatory agents, and in the maintenance of mitochondrial function, cellular redox, and protein homeostasis 1. Nrf2 protein comprises seven functional domains termed Nrf2‐ECH homology (Neh) 1–7 domains (Fig. 1). Nrf2 binds one of its major negative regulators, Kelch‐like ECH‐associated protein 1 (Keap1) through its N‐terminal Neh2 domain. Neh4 and Neh5 are transactivation domains that recruit cAMP response element‐binding protein (CREB)‐binding protein (CBP) and/or receptor‐associated coactivator 3 (RAC3). The Neh7 domain mediates binding to retinoid X receptor alpha (RXRα), another negative regulator of Nrf2. The Neh6 domain mediates interaction with a third negative regulator, β‐transducin repeat‐containing protein (β‐TrCP). Neh1 is responsible for the formation of a heterodimer with small musculoaponeurotic fibrosarcoma (sMaf) proteins, and mediates binding to antioxidant/electrophile response element (ARE/EpRE) sequences in the promoter regions of Nrf2 target genes. Finally, the C terminus Neh3 is another transactivation domain that recruits chromo‐ATPase/helicase DNA‐binding protein 6 (CHD6).

**Figure 2:**
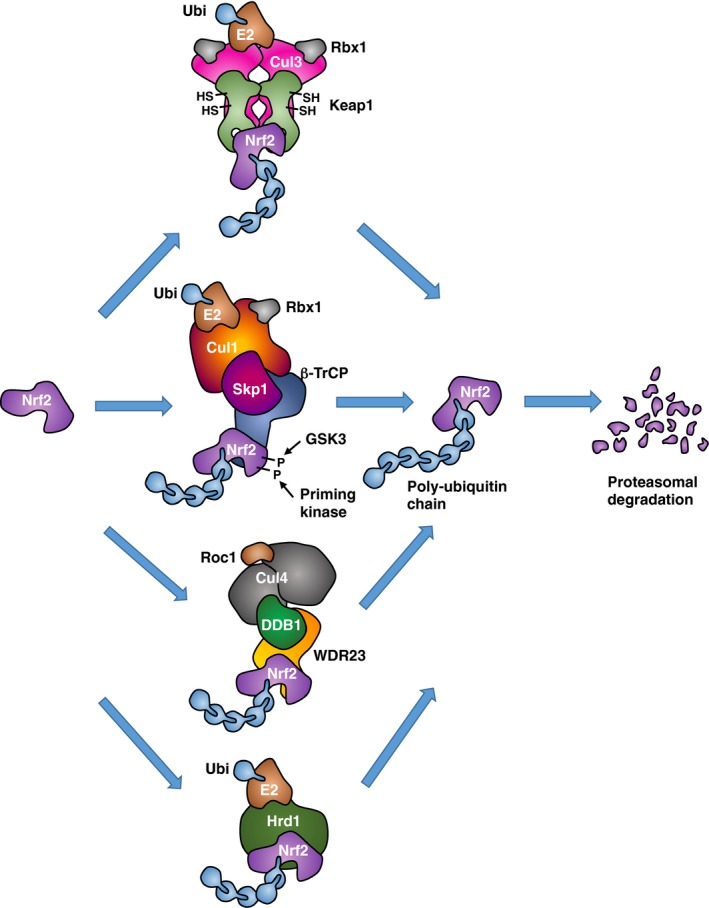
Regulation of Nrf2 by ubiquitination and proteasomal degradation. Four known ubiquitin ligase systems mediate the degradation of Nrf2: Keap1, a substrate adaptor protein for Cul3/Rbx1‐based ubiquitin ligase and a cysteine‐based sensor for Nrf2 inducers; β‐TrCP, a substrate adaptor for Skp1‐Cul1/Rbx1‐based ubiquitin ligase, WDR23‐DDB1‐Cul4//Roc1‐based ubiquitin ligase, and Hrd1. Keap1‐mediated degradation requires that its cysteine sensors are in reduced state. β‐TrCP‐mediated degradation requires formation of a phosphodegron catalyzed by glycogen synthase kinase 3 (GSK3), which in turn requires phosphorylation by a priming kinase. Hrd1‐mediated degradation of Nrf2 occurs during ER stress.

Nrf2 levels are regulated primarily by ubiquitination and proteasomal degradation (Fig. 2). After binding to the Neh2 domain, Keap1 mediates the Cullin3 (Cul3)/Rbx1‐dependent ubiquitination of Nrf2 2, 3, 4. Additionally, the Neh6 domain contains a phosphodegron for β‐TrCP/Cullin1‐mediated ubiquitination 5, 6. Synoviolin (Hrd1) and WDR23‐DDB1‐Cul4 are two other ubiquitin ligases that have been shown to participate in the proteasomal degradation of Nrf2 7, 8.

At homeostatic conditions, Nrf2 is a short‐lived protein. Under stress conditions, Nrf2 is stabilized and translocates to the nucleus, where it binds (as a heterodimer with a member of the small Maf family of transcription factors) to the ARE/EpRE sequences in the promoter of its target genes, and activates their transcription. Nrf2 targets include genes that encode detoxification, antioxidant, and anti‐inflammatory proteins as well as proteins involved in the regulation of autophagy and clearance of damaged proteins, such as proteasomal subunits 9, 10, 11. Activation of Nrf2 leads to the upregulation of proteins involved in the synthesis of glutathione, the main intracellular small molecule antioxidant 12, 13, and NADPH, which provides reducing equivalents for the regeneration of reduced glutathione (GSH) from its oxidized form, GSSG 14, 15, 16, 17. Nrf2 also participates in the maintenance of mitochondrial function 18, 19 and quality control, through activation of mitophagy 20, 21. Nrf2 inhibits the transcription of genes encoding proinflammatory cytokines and suppresses proinflammatory responses following exposure to ultraviolet radiation 22 or lipopolysaccharide (LPS) 23. Such comprehensive cytoprotective functions suggest potential benefits of therapeutic targeting of Nrf2 to counteract neurodegeneration.

## Nrf2 in neurodegenerative diseases

Aging is the primary risk factor for developing neurodegenerative diseases 24. The prevalence of age‐dependent neurodegenerative disorders, such as Alzheimer's disease (AD) and Parkinson's disease (PD), is projected to increase due to population aging 25, with a corresponding increase in the associated socioeconomic impact. Genetic and environmental risk factors also contribute to the development of neurodegenerative diseases. Currently, there is no disease‐modifying therapy for any neurodegenerative disease; and even in conditions where symptomatic treatments are available, the therapeutic benefit is limited. Although the clinical manifestations and genetic causes of neurodegenerative diseases are distinct, several of these disorders share strikingly similar pathological mechanisms, including mitochondrial impairment, excessive levels of reactive oxygen species (ROS), neuroinflammation, and disturbances in protein homeostasis (proteostasis) 24. Additionally, there is an age‐associated increase in oxidative damage to the brain, leading to mitochondrial dysfunction, dopamine auto‐oxidation, α‐synuclein aggregation, glial cell activation, alterations in calcium signaling, and excess free iron 26. Dopaminergic neurons show linear decline of 5–10% per decade with aging, and this process is accelerated in PD patients. A meta‐analysis of PD and AD microarray datasets identified 31 common downregulated genes containing the ARE/EpRE consensus sequence in their promoters, in addition to increased levels of Nrf2 27. Increased oxidative stress was observed in human peripheral blood mononuclear cells isolated from individuals with mild cognitive impairment and from 3‐month‐old 3xTg‐AD male mice, which was due to increased levels of nuclear Nrf2 and reduced superoxide dismutase 1 (SOD1) mRNA in the brain cortex 28. The AT‐Nrf2‐knockout mouse model, that combines amyloidopathy and tauopathy with Nrf2 deficiency, presents increased markers of oxidative stress and neuroinflammation in the brain tissue compared to AT‐Nrf2‐wild‐type mice 29. Furthermore, young adult AT‐Nrf2‐knockout mice have deficits in spatial learning and memory and reduced long‐term potentiation in the perforant pathway. Transcriptomic analysis has shown that Nrf2‐knockout mouse brains share 7 and 10 of the most dysregulated pathways with aging human and AD brains, respectively 29.

## Oxidative stress in neurodegenerative disease and the counteracting role of Nrf2

The brain is especially sensitive to changes in cellular redox status; therefore, maintaining redox homeostasis in the brain is critical for preventing oxidative stress‐induced cellular damage 24, 25. Nrf2 regulates the expression of a variety of antioxidant enzymes and proteins that exert cytoprotective effects, and there is mounting evidence demonstrating improvement of neurological phenotypes in disease models following the induction of Nrf2‐dependent antioxidant activities.

Synaptic degeneration and neuronal death that characterize AD are caused by several deregulated processes including: (a) increased oxidative stress, (b) chronic inflammation (c) mitochondrial dysfunction, (d) accumulation of amyloid‐β (Aβ) 1–42 peptides, generated from the amyloid precursor protein (APP), (e) proteasome inhibition, (f) mutations in APP, presenilin‐1 and presenilin‐2 genes, and (g) aggregation of hyper‐phosphorylated tau protein 30. Oxidative stress is, at least partially, induced by oligomeric Aβ peptides, and precedes other biochemical changes, while oxidative damage in turn induces chronic inflammation.

**Figure 3:**
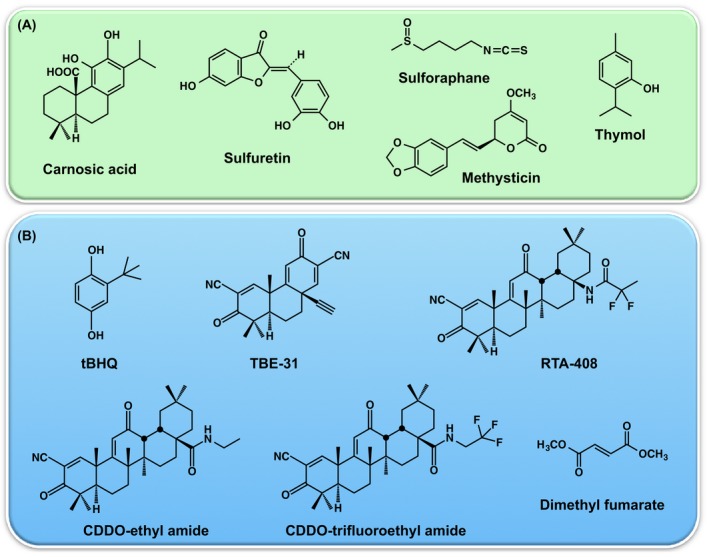
Chemical structures of naturally occurring (A) and synthetic (B) Nrf2 activators with demonstrated efficacy in models of neurological conditions.

Carnosic acid (Fig. 3) is a natural pro‐electrophilic compound that is converted to its active form by oxidative stress, which in turn activates Nrf2‐dependent transcription 31. *In vitro*, carnosic acid reduced dendritic spine loss in rat neurons exposed to neurotoxic oligomeric Aβ. *In vivo*, carnosic acid treatment of human amyloid precursor protein (hAPP)‐J20 mice improved learning and memory. Histological analysis showed that carnosic acid increased dendritic and synaptic markers, and decreased astrogliosis, Aβ plaque number, and phospho‐tau staining in the hippocampus. Sulfuretin (Fig. 3), a flavonoid glycoside, significantly attenuated the decrease in cell viability and accumulation of ROS associated with Aβ25‐35‐induced neurotoxicity in neuronal cells 32. Sulfuretin stimulated the activation of Nrf2, resulting in increased expression of the antioxidant Nrf2‐target gene heme oxygenase‐1 (HO‐1). The neuroprotective effects of sulfuretin were diminished following Nrf2 small interfering RNA (siRNA) injection and the addition of HO‐1 inhibitor zinc protoporphyrin IX 32.

Parkinson's disease is characterized by progressive loss of dopaminergic neurons and profound reduction of dopamine in the striatum. Although multiple genetic and environmental factors have been implicated in the etiology of PD, the majority of the clinical cases are sporadic. The 1‐methyl‐4‐phenyl‐1, 2, 3, 6‐tetrahydropyridine (MPTP) mouse model of PD recapitulates the effects of the neurotoxin MPTP‐induced Parkinsonism in humans. Nrf2 activation showed neuroprotective effects in MPTP mice, and these were associated with a reduction of oxidative damage and neuroinflammation 33, 34, 35, 36. Notably, astrocytes from adult and old rats are more susceptible to MPP+ toxicity than astrocytes from newborn rats, but following pretreatment with the Nrf2 activator *tert*‐butyl‐hydroquinone (Fig. 3), Nrf2‐dependent expression of antioxidant enzymes is increased, resulting in cytoprotection 37.

Huntington's disease (HD) is an autosomal dominant and highly penetrant neurodegenerative disorder, which results from the pathological expansion of trinucleotide CAG repeats – encoding polyglutamine – in the huntingtin (HTT) protein 38. At the time of diagnosis, brains from HD patients show marked striatal and cortical atrophy. Although the pathogenesis of HD is complex, oxidative stress is an important driver of pathology. Compared to nondisease controls, the levels of Nrf2 targets, including glutathione peroxidase, catalase, and SOD1, are increased in human HD brains 39. This suggests a partial activation of the Nrf2‐dependent antioxidant defense. Based on studies using various animal models, pharmacological Nrf2 activation holds promise for combating oxidative stress in HD.

## Role of Nrf2 in mitochondrial function

Nrf2 regulates cellular defense through the modulation of mitochondrial function. Nrf2 induction counterbalances mitochondrial production of ROS and defends against mitochondria‐produced toxins 19, 40. Nrf2 function is decreased in mitochondria‐related disorders, such as AD, PD, and Friedreich's ataxia (FRDA). Studies using isolated mitochondria and cultured cells have demonstrated that Nrf2 deficiency leads to impaired mitochondrial fatty acid oxidation, respiration, and ATP production 18, 41. Chemical activators of Nrf2 promote mitochondrial integrity either by inducing mitophagy 20, 21 or by conferring resistance to the oxidative stress‐mediated opening of the mitochondrial permeability transition pore 42, 43. Emerging evidence suggests that Nrf2 also influences mitochondrial biogenesis, particularly under stress conditions. Nrf2 activates proliferator‐activated receptor gamma coactivator 1α (PGC‐1α) and nuclear respiratory factor 1, thereby stimulating mitochondrial biogenesis 44. A recent report has shown that increased proteasomal activity, one of the consequences of Nrf2 activation, leads to enhanced degradation of the mitochondrial fission protein dynamin‐related protein 1 (Drp1), promoting mitochondrial hyperfusion 45. The lower levels of Drp1 under conditions of Nrf2 activation could be beneficial in AD as partial reduction of Drp1 decreases the levels of phosphorylated Tau, a major microtubule‐associated protein (MAPT) of mature neurons, and improves mitochondrial dynamics and synaptic activity in Tau transgenic (P301L) mice 46.

Mitochondrial mass and oxygen consumption increase in primary glial cultures following treatment with the α7 acetylcholine nicotinic receptor (nAChR) agonist PNU282987 in the absence of oxidative stress 47. Importantly, these changes were abolished when either Nrf2 was absent, HO‐1 was inhibited, or PGC‐1α was silenced. Moreover, microglia of PNU282987‐treated animals showed a significant increase in mitochondrial content, while in HO‐1 mutant (LysMcre‐Hmox1Δ/Δ) and PGC‐1α‐deficient strains, the animals showed lower mitochondrial levels in microglia and treatment with PNU282987 was ineffective. These results suggest that α7 nAChR signaling increases glial mitochondrial mass via Nrf2‐dependent stimulation of HO‐1 and PCG‐1α.

Transcriptomic and metabolomic analyses of mutant *Drosophila* deficient for the mitochondrial serine/threonine‐protein kinase PTEN‐induced putative kinase 1 (PINK1), a model of PD, showed that PINK1 deficiency leads to alterations in nucleotide metabolism, suggesting that enhancing nucleotide biosynthetic pathways could be a strategy to reverse mitochondrial dysfunction in PD 48. Activation of Nrf2 increases the glucose flux through the pentose phosphate pathway and affects the metabolism of folate and glutamine, which in addition to upregulating NADPH and GSH biosynthesis (mentioned above), results in enhanced purine biosynthesis 17. Thus, enhancing nucleotide biosynthesis via Nrf2 activation might reverse mitochondrial dysfunction in PINK1 deficiency. This idea is supported by experiments showing that pharmacological activation of Nrf2 in PINK1‐knockout cells restores the mitochondrial membrane potential (∆ψ_m_) and protects against dopamine toxicity 49.

Friedreich's ataxia is an autosomal recessive neurodegenerative disorder, caused by the expansion of intronic GAA repeats resulting in reduced levels of the mitochondrial protein frataxin – an essential protein for the biogenesis of iron‐sulfur clusters and proper functioning of mitochondrial respiratory chain complexes 50. Several studies using mouse models and cultured cells from patients with FRDA, have reported impaired Nrf2 signaling in FRDA 50, 51, 52. The sensitivity to oxidative stress of YG8R and KIKO fibroblasts from FRDA mouse models can be prevented by Nrf2 inducers, such as the isothiocyanate sulforaphane (Fig. 3) and the tricyclic cyanoenone TBE‐31 (Fig. 3) 53. The pentacyclic cyanoenone RTA‐408 (Omaveloxolone) (Fig. 3) is currently in a Phase 2 clinical trial in FRDA patients (ClinicalTrials.gov). Interestingly, Nrf2 binds an upstream response element in the frataxin locus, and the anesthetic dyclonine has been shown to activate Nrf2, increase the mRNA and protein levels of frataxin and rescue frataxin‐dependent enzyme deficiencies in the iron‐sulfur enzymes aconitase and succinate dehydrogenase 54.

## Role of Nrf2 in neuroinflammation

A bidirectional communication takes place between the brain and the peripheral immune system in both physiological and pathological conditions 55. Brain inflammation results from acute injury or following the accumulation of mutant proteins or endogenous neurotoxic metabolites such as those associated with neurodegenerative diseases, including AD and PD. Microglia, in particular, play a key role in brain inflammation via the release of proinflammatory cytokines. Increased neuroinflammation and oxidative stress following microglial activation are associated with age‐related cognitive impairment 55, 56.

The anti‐inflammatory properties of Nrf2 signaling are well established, and recent evidence suggests a mechanism of transcriptional repression of proinflammatory cytokines (TNF‐α, IL‐1, IL‐6, IL‐8, MCP‐1) in microglia, macrophages, monocytes, and astrocytes following Nrf2 activation 23, 57. The Nrf2 activator sulforaphane increased Nrf2 DNA‐binding activity and upregulated Nrf2 target genes in RAW264.7 cells, BV2 microglia cells and primary mouse microglia, while reducing LPS‐induced interleukin IL‐1β, IL‐6, and inducible nitric oxide synthase (iNOS) 58, 59, 60. Furthermore, representatives of seven distinct chemical classes of Nrf2 activators show highly correlated upregulation of NQO1 – a prototypic Nrf2 target – and suppression of iNOS and COX‐2 expression in cell lines and primary mouse peritoneal macrophages 61, 62.

Oral administration of the kavalactone methysticin (Fig. 3) activated the Nrf2 pathway in the hippocampus and cortex of AD (APP/Psen1) mice 63. This treatment reduced microgliosis, astrogliosis, and the secretion of the proinflammatory cytokines TNF‐α and IL‐17A, as well as oxidative damage. Most importantly, the long‐term memory decline of AD mice was significantly attenuated.

Several human studies have demonstrated the protective effects of n‐3 polyunsaturated fatty acids (n‐3 PUFA), particularly in the early stages of mild cognitive impairment preceding AD 64. The Nrf2‐dependent anti‐inflammatory and antioxidant effects of n‐3 PUFA have been linked to a lower activation of microglia. It has been suggested that n‐3 PUFA shift microglia from the macrophage M1 to an M2 phenotype, resulting in lower oxidative stress and enhanced phagocytic activity toward Aβ peptide 64.

Nrf2 signaling‐mediated oxidative stress and neuroinflammation have also been implicated as the key therapeutic targets for amyotrophic lateral sclerosis (ALS). Genetic studies in ALS mouse models have shown a significant therapeutic effect of elevated Nrf2 levels in astrocytes, the major GSH suppliers for neighboring neurons 65. Moreover, Nrf2 signaling is critical for attenuating neuroinflammation in ALS through repression of the deleterious effects of activated microglia on neuronal survival. Consistent with the therapeutic potential of Nrf2 signaling, treatment with small molecule activators, including the potent cyanoenone triterpenoids, has shown efficacy in mouse models of ALS 66.

## Role of Nrf2 in autophagy

Autophagy recycles macromolecular aggregates, resulting from oxidative stress, and may also reduce the mitochondrial production of ROS through recycling of old and damaged mitochondria 67, 68. The deregulation of autophagy initiation or progression results in the accumulation of aggregation‐prone proteins in neuronal and extraneural tissues.

Nrf2 promotes autophagy via induction of the autophagosome cargo protein p62/sequestosome‐1 (p62/SQSTM1) 69, 70 and other autophagy‐related genes 10. Knockdown of Nrf2 reduces autophagic flux, whereas pharmacological Nrf2 activation promotes autophagy, and this closely correlates with changes in the levels of p62/SQSTM1 69, 70. This area of research is of particular importance given the strong association between neurological phenotypes and the presence of accumulated misfolded proteins in affected brains, and deficiency of the protein degradation machinery in neurodegenerative diseases. The role of Nrf2 in the clearance of misfolded proteins has received much attention in recent studies, and the relationship with other Nrf2‐controlled stress responses has been evaluated in neurodegenerative and healthy conditions.

Redox mechanisms of autophagy regulation have been documented at the level of crosstalk between the Nrf2 pathway and p62‐associated autophagy. In a *Drosophila* model of FRDA, genetic or pharmacologic inhibition of the mechanistic target of rapamycin complex 1 (TORC1) signaling led to an increase in the cellular levels of ATP and organismal lifespan, which was in part due to enhanced transcription of antioxidant genes mediated by the *Drosophila* ortholog of Nrf2 71. Notably, under condition of oxidative stress, the protective effect of TORC1 inhibition was abolished upon inhibition of autophagy.

A recent study has demonstrated that nine autophagy genes containing ARE/EpRE sequence are responsive to Nrf2 activation by sulforaphane in human and mouse cells 10. Mouse embryonic fibroblasts from Nrf2‐knockout mice have reduced expression of autophagy genes, and this deficiency can be rescued by an Nrf2‐expressing lentivirus 10. Compared to their wild‐type counterparts, the neuronal levels of p62/SQSTM1, CALCOCO2/NDP52, ULK1, ATG5, and GABARAPL1 are low in Nrf2‐deficient mice co‐expressing mutant APP (HsAPPV717I) and MAPT (HsMAPTP301L), and these animals have more intracellular aggregates of the mutant proteins. Additionally, in the absence of Nrf2, the colocalization of the mutant APP and MAPT proteins with p62/SQSTM1 was impaired. The same study showed that in AD patient samples, neurons expressing high levels of APP or MAPT also have high levels of nuclear Nrf2 and its transcriptional target p62/SQSTM1 10.

A hallmark of the AD brain is the presence of senile plaques containing insoluble amyloid‐β peptide, a cleavage product of the APP, which is physiologically degraded by autophagy. The neurotoxic amyloid‐β peptide is the result of the enzymatic activity of β and/or γ secretase. The effect of genetic removal of Nrf2 has been investigated in double transgenic mice expressing a chimeric mouse/hAPP (Mo/HuAPP695swe) and a mutant human γ secretases (presenilin 1 (PS1‐dE9) 72. Compared to Nrf2‐proficient animals, APP/PS1 mice that are also deficient for Nrf2 had higher intracellular levels of APP, Aβ (1–42), and Aβ (1–40), which was accompanied by enhanced inflammation. In these Nrf2 mutant mice, the levels of poly‐ubiquitin‐conjugated proteins were increased, APP and Aβ were primarily found in the insoluble fraction, and autophagy was impaired – as evidenced by accumulation of multivesicular bodies, endosomes, and lysosomes.

In certain cases of PD, rare mutations in leucine‐rich repeat kinase 2 (LRRK2) and α‐synuclein (α‐Syn) lead to neurodegeneration, associated with accumulation of misfolded proteins 73, 74. Using a longitudinal imaging platform, the fate and location of mutant LRRK2 and α‐Syn were recently investigated in single live neurons 75. Activation of Nrf2 protected against the toxicity of the mutant proteins in a cell‐autonomous and time‐dependent manner. Interestingly, the protective effect of Nrf2 was different depending on the mutant proteins: Nrf2 activation led to a decrease in the steady‐state levels of α‐Syn by increasing its degradation, whereas misfolded diffuse LRRK2 was sequestered into inclusion bodies 75.

Genetic studies have identified rare mutations in *SQSTM1* gene, which causes susceptibility to frontotemporal lobar degeneration (FTLD) and ALS, which further emphasizes a relationship between Nrf2 and autophagy. The term FTLD describes a spectrum of neurodegenerative disorders, characterized, by the deposition of misfolded proteins and focal atrophy of frontal and/or temporal lobes 76. Mutations in genes encoding three autophagy adaptor proteins, p62/SQSTM1, ubiquilin 2, and optineurin, represent a known causative factor for FTLD, implicating autophagy in FTLD pathogenesis. In the majority of FTLD cases, the accumulated protein aggregates contain either microtubule‐associated protein tau or TAR DNA‐binding protein (TDP)‐43 77. Intracellular deposition of TDP‐43 is characterized by prominent neuronal granular cytoplasmic immunoreactivity and abundant oligodendroglial inclusions 78. Aggregates are also positive for ubiquitin and p62/SQSTM1, indicating that these aggregates are targeted for degradation 79. During autophagy, the recognition of the lipidated form of microtubule‐associated protein light chain 3 isoform B (LC3B‐II) within the phagophore membrane by p62/SQSTM1 represents a critical protein–protein interaction that is mediated through its LC3‐interacting region, and notably, some FTLD and ALS mutations of p62/SQSTM1 map to this region, leading to reduction in LC3B binding affinity and limiting the recruitment of p62/SQSTM1 to the phagophore 80.

Mutations in *SQSTM1* have been found in patients with ALS, FTLD or with FTLD/ALS when both syndromes are present in the same person 81. Analysis of 348 ALS and FTLD missense mutations in 14 genes focusing on protein stability based on available 3D structures predicted that most of the missense mutations with destabilizing energies occur in the regions that control protein–protein interactions, and that the predicted destabilization is greater for ALS compared to FTLD mutations and correlates with disease progression. However, other ALS causative mutations, which span throughout the *SQSTM1* gene, have been identified, namely E81K, N239K, G297S, E372D, P388S, and P392L, suggesting more complex functions of the protein in disease pathogenesis 82. Intriguingly, some *SQSTM1* mutations are associated with reduced Nrf2 activation in FTLD/ALS 83, with pharmacological Nrf2 activators showing beneficial effects 84.

## Nrf2 signaling in aging

Although some of the downstream biochemical pathways regulated by Nrf2 are directly implicated in neurodegeneration, it is important to evaluate the role of Nrf2 and its impact on the aging processes in the brain as a conceptual paradigm. Aging is tightly associated with redox events, and the free radical theory of aging indicates that redox imbalance may be a promoting factor. A study using *Caenorhabditis elegans* and human fibroblasts compared differential responses to oxidative stress challenge in young and old individuals 85. In young individuals, higher levels of ROS were generated, and signaling pathways, including p‐ERK, p‐AKT, and p‐AMPKα/β, were activated in response to oxidative stress. Nrf2 translocated to the nucleus and induced the expression of antioxidant and detoxification enzymes, including SOD1, CAT, GPX, HO‐1, GSTP‐1, to maintain redox homeostasis. Moreover, young individuals had a better capacity to degrade damaged proteins by upregulating the expression of molecular chaperones and enhancing the activity of the proteasome 85.

Compelling evidence for Nrf2 suppression in aging came from a study of Hutchinson‐Gilford progeria syndrome (HGPS), a rare fatal premature aging disorder 86. The cause for the disease is progerin, a mutant form of the nuclear architectural protein lamin A. The constitutive production of progerin leads to morphological, genomic, and epigenetic changes, and to mesenchymal stem cell attrition. The study found that progerin sequesters Nrf2, resulting in subnuclear mislocalization and impaired activity of Nrf2 leading to chronic oxidative stress. The aging defects that occur in HGPS fibroblasts can be recapitulated by Nrf2 knockdown or increased oxidative stress caused by treatment with hydrogen peroxide of wild‐type fibroblasts. Conversely, activation of Nrf2 by genetic or pharmacological (by oltipraz treatment) means reverses the nuclear aging defects in HGPS patient cells and restores the viability of HGPS mesenchymal stem cells implanted into the tibialis anterior muscle of immunodeficient mice. These findings strongly suggest that repression of Nrf2 signaling is an essential contributor to the premature aging phenotype.

The regenerative function of neural stem/progenitor cells (NSPCs) continuously declines with advancing age. This specific temporal pattern of NSPC decline is functionally relevant at a behavioral level and correlates with decreased levels of Nrf2 87. Moreover, newborn Nrf2‐knockout mice exhibit a lower number of NSPCs in comparison with their wild‐type counterparts, and the proliferative and neurogenic potential of these NSPCs is compromised in these Nrf2‐deficient animals.

The age‐dependent decline in stem cell proliferation is most likely due to dysregulated signaling in the neurogenic niche and increased levels of inflammatory cytokines 88. Treatment with NT‐020, a proprietary blend of polyphenols, led to an increase in nuclear localization of Nrf2 and expression of its downstream transcriptional target HO‐1 in all subsets of cell types in the subgranular zone of the dentate gyrus and the subventricular zone in brains of both young and aged rats. Analyses of hippocampal tissue showed that the levels of a number of proinflammatory factors, including TNF‐α and IL‐1β, were reduced, whereas the levels of the anti‐inflammatory cytokines IL‐24, IL‐4, and IL‐10 increased. Furthermore, NT‐020 treatment improved performance on a spatial learning task in aged rats 89.

An important question is which aging brain cells lose their Nrf2 activation responses? Markers of Nrf2 activity can be observed in neurons in postmortem brain tissue and animal models of disease; however, recent evidence demonstrates that Nrf2 activity is low in iPS‐derived human neurons 90, and that during mouse cortical neuronal development *in vitro* and *in vivo*, the expression of Nrf2 is repressed by epigenetic inactivation of its promoter 91. In contrast, non‐neuronal cells such as astrocytes demonstrate consistent *in vitro* and *in vivo* Nrf2 activation responses 92. Astrocytes are highly dynamic cells that have roles in the maintenance of brain homeostasis and energy metabolism, regulation of neurotransmission, and processing of synaptic information and inflammatory triggers 93. One essential function of astrocytes is to provide antioxidant support to neighboring neurons 94. Consistently with their critical role, an astrocyte‐specific, but not neuronal‐specific, Nrf2‐activation rescued the neurological phenotype of mutant SOD1 mouse model of ALS 95. Astrocytes show an age‐dependent increase in oxidative/nitrosative stress, mitochondrial dysfunction, RNA oxidation, NADPH oxidase activity, superoxide levels, and iNOS expression. Astrocytes also display age‐dependent inflammatory response with higher levels of proinflammatory cytokines, such as TNF‐α, IL‐1β, IL‐6, IL‐18, as well as increased mRNA levels of cyclooxygenase 2 (COX‐2) 93. Nevertheless, aged astrocytes are amenable for Nrf2 activation and represent a target cell type for therapeutic intervention 37.

Long‐term consumption of high fat diet (HFD) contributes to cognitive impairment in aged mice 96. A 16‐week HFD increased age‐related oxidative damage (protein carbonyls) in the brain and impaired cognitive performance in behavioral tests of 20‐month‐old male mice. This selective increase in oxidative damage and cognitive decline was associated with decreased levels and activity of Nrf2, suggesting the involvement of lower antioxidant responses. In contrast to HFD consumption, moderate caloric restriction (CR) confers antioxidative and anti‐inflammatory effects, preserving a youthful phenotype in rodents 97. CR prevents age‐related increases in oxidative stress and inflammation and age‐related dysfunction of Nrf2. Intriguingly, exposure of aged cells to resveratrol, a CR mimicking compound and an activator of the cellular energy sensor AMP‐activated protein kinase (AMPK) 98, results in rapid activation of Nrf2 signaling 99, consistent with early reports on the ability of this compound to induce drug metabolizing enzymes and inhibit inflammation 100. Oral administration of another natural product, thymol (Fig. 3), a monoterpene phenol, has been shown to exhibit neuroprotective effects in C57BL/6J mice fed a HFD for 12 weeks 101. Thymol treatment significantly reversed the gain of body weight, improved cognitive impairments and decreased HFD‐induced Aβ deposition and tau hyperphosphorylation in the hippocampus, and these protective effects of thymol were associated with activation of Nrf2 signaling.

Chronic administration of testosterone propionate to aged rats has been shown to ameliorate the decline of balancing reactions and muscular strength associated with aging 102. Interestingly, these beneficial effects are also correlated with decreased oxidative stress, increased protein levels of Nrf2 and its transcriptional targets HO‐1 and NQO1, and greater number of tyrosine hydroxylase immunoreactive cells in substantia nigra, implicating Nrf2 as an important mediator of some of the physiological and behavioral effects of testosterone.

## Nrf2‐dependent amelioration of age‐dependent neurological phenotype

A number of pharmacological Nrf2 activators are currently in clinical trials, and the fumaric acid ester dimethylfumarate (Fig. 3) is an FDA‐approved drug (Tecfidera, Biogen‐Idec) for the treatment of relapsing multiple sclerosis (MS). Dimethylfumarate blocks the degradation of Nrf2 by covalent modification of cysteine 151 in Keap1 103, which in addition to serving as a substrate adaptor for Cul3‐mediated degradation for Nrf2, acts as the protein sensor for electrophilies and oxidants 104. Following stereotaxic delivery of recombinant adeno‐associated viral vector expressing human α‐synuclein to the ventral midbrain of mice, daily oral administration of dimethylfumarate protected nigral dopaminergic neurons against the toxicity of α‐synuclein, and decreased astrocytosis and microgliosis; notably, this protective effect was not observed in Nrf2‐knockout mice 105. Dimethylfumarate is, however, of a relatively low potency and specificity. Other molecules with similar mechanism of action or with the ability to directly disrupt the Keap1/Nrf2 interaction are emerging as potential protective agents for the prevention and treatment of a broad range of currently incurable neurodegenerative diseases 57, 59, 106. Inhibition of Keap1 has also been shown to prevent neuronal toxicity in response to the AD‐initiating Aβ42 peptide, in correlation with Nrf2 activation 106. Consistent with this notion, pharmacological activation of Nrf2 by the cyanoenone triterpenoids CDDO‐ethyl amide and CDDO‐trifluoroethyl amide (Fig. 3) induces broad antioxidant effects in HD mouse brain and ameliorates the neurological phenotype 107. The benefits of Nrf2 activation can be extended to other polyglutamine diseases such as spinal and bulbar muscular atrophy (SBMA, also known as Kennedy's disease) 108.

## Concluding remarks

**Figure 4:**
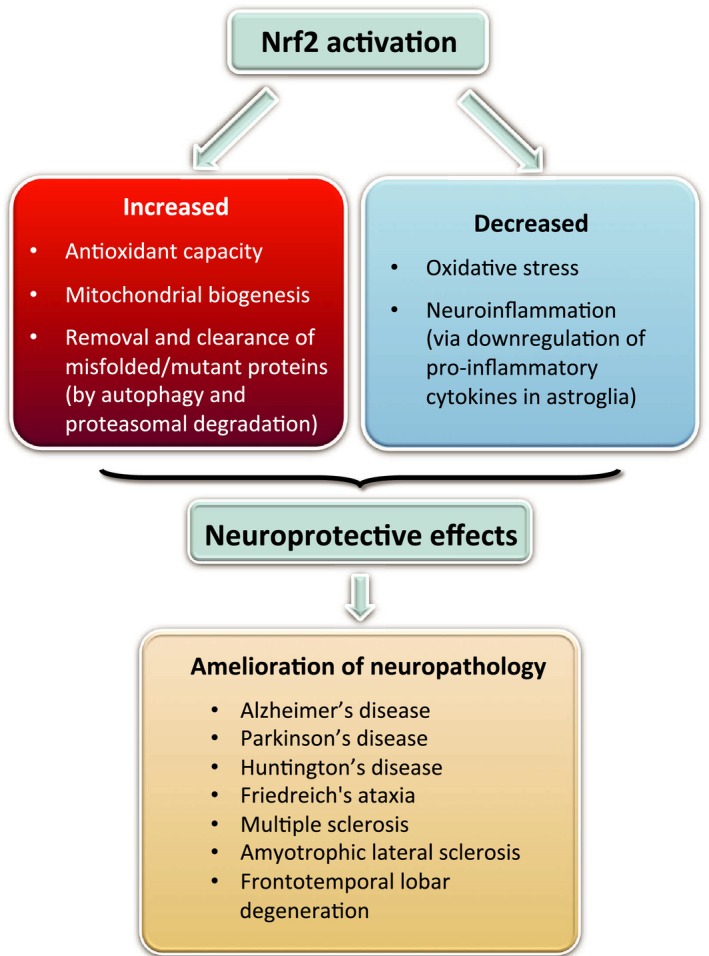
The multiple neuroprotective effects of Nrf2 and human pathologies, in which Nrf2 activation is envisioned to be therapeutically beneficial.

In summary, it is becoming increasingly clear that Nrf2 activation counteracts multiple pathogenic processes involved in neurodegenerative disorders through upregulation of antioxidant defenses, inhibition of inflammation, improvement of mitochondrial function, and maintenance of protein homeostasis (Fig. 4). Finally, it is worthwhile pointing out that there appears to be a notable inverse relationship between the expression of mutant proteins that are causally associated with a number of neurodegenerative diseases and Nrf2‐dependent cytoprotective responses. Increased nuclear translocation of Nrf2 is found in a mutant transactive response DNA‐binding protein 43 (TDP‐43) transgenic model of ALS, but the expression of downstream antioxidant enzymes is decreased 109. A gene expression profiling has revealed an upregulation of Nrf2‐target gene expression in the spinal cord of TDP‐43Q331K mice compared to their control counterparts; however, the corresponding protein levels were not increased 110. In TDP‐43M337V patient fibroblasts and astrocyte cell cultures from TDP‐43Q331K mice, the levels of glutathione are low even though the mRNA levels of antioxidant enzymes are high 110. An increase of nuclear Nrf2 in brain cortex, but a decreased expression of Nrf2‐responsive targets was reported in an AD mouse model 28. The activation of Nrf2 by proteasomal inhibition is inhibited in cells expressing PINK1 G309D 111, a mutant PINK1 causally associated with hereditary early‐onset PD 112. The repressive effects of mutant HTT on Nrf2 responses have been observed in immortalized murine striatal cells expressing mutant HTT 113 and in human HD neural stem cells 57. Importantly, pharmacological Nrf2 activation, which was essentially absent in HD neural stem cells with an extreme (72) CAG repeat length, was restored upon isogenic genetic correction of the CAG expansion to a nonpathological (21) CAG repeat length 57. Collectively, these findings suggest a negative effect of a number of mutant proteins across a large spectrum of neurodegenerative diseases on functional Nrf2‐dependent responses and thus a possible exacerbation of the neurological phenotype through reduced Nrf2 signaling. Nonetheless, there is a growing body of convincing experimental evidence demonstrating that pharmacological Nrf2 activation is beneficial in counteracting many of the pathological processes that occur in neurodegenerative diseases, which although of distinct clinical manifestations, share similar molecular mechanisms. Thus, targeting Nrf2 signaling by pharmacological entities, some of which are currently in clinical trials, may provide a therapeutic option to delay onset, slow progression, and ameliorate symptoms of neurodegenerative disorders, including clinical cases of unknown etiology.

## Author contributions

ATD‐K and AGK discussed and developed the concept of the review. AGK wrote the first draft. RVK prepared the figures. All authors participated in writing and editing of the manuscript.
